# Genomic Characterization of *Campylobacter jejuni* Strain M1

**DOI:** 10.1371/journal.pone.0012253

**Published:** 2010-08-26

**Authors:** Carsten Friis, Trudy M. Wassenaar, Muhammad A. Javed, Lars Snipen, Karin Lagesen, Peter F. Hallin, Diane G. Newell, Monique Toszeghy, Anne Ridley, Georgina Manning, David W. Ussery

**Affiliations:** 1 Department of Systems Biology, The Technical University of Denmark, Lyngby, Denmark; 2 Molecular Microbiology and Genomics Consultants, Zotzenheim, Germany; 3 School of Science and Technology, Nottingham Trent University, Nottingham, United Kingdom; 4 Department of Chemistry, Biotechnology and Food Sciences, Norwegian University of Life Sciences, Ås, Norway; 5 Institute of Medical Microbiology, Oslo University Hospital, Oslo, Norway; 6 Department of Informatics, University of Oslo, Oslo, Norway; 7 Veterinary Laboratories Agencies, Addlestone, United Kingdom; 8 Food-borne Zoonoses Consultancy, Andover, United Kingdom; Miami University, United States of America

## Abstract

*Campylobacter jejuni* strain M1 (laboratory designation 99/308) is a rarely documented case of direct transmission of *C. jejuni* from chicken to a person, resulting in enteritis. We have sequenced the genome of *C. jejuni* strain M1, and compared this to 12 other *C. jejuni* sequenced genomes currently publicly available. Compared to these, M1 is closest to strain 81116. Based on the 13 genome sequences, we have identified the *C. jejuni pan*-genome, as well as the core genome, the auxiliary genes, and genes unique between strains M1 and 81116. The pan-genome contains 2,427 gene families, whilst the core genome comprised 1,295 gene families, or about two-thirds of the gene content of the average of the sequenced *C. jejuni* genomes. Various comparison and visualization tools were applied to the 13 *C. jejuni* genome sequences, including a species pan- and core genome plot, a BLAST Matrix and a BLAST Atlas. Trees based on 16S rRNA sequences and on the total gene families in each genome are presented. The findings are discussed in the background of the proven virulence potential of M1.

## Introduction

*Campylobacter jejuni* is the most common cause of known bacterial enteritis in Europe and in the US, and the second-most cause (following *Salmonella* infections) in many other countries [1], [2]. Campylobacteriosis is often food-transmitted, and frequently attributed to the consumption and handling of poultry meat [3]. Chickens are commonly asymptomatically colonized with *C. jejuni*, and, depending on the season and country, 20% to 100% of flocks can be found positive at slaughter [4]. Contamination of carcasses occurs during processing, so that up to 80% of retail poultry meat is positive for *Campylobacter* in countries like the UK [5], [6].

There is considerable circumstantial evidence from epidemiological studies, like case control investigations, that poultry is a major attributable source of human infection with *C. jejuni*. Molecular epidemiological investigations also provide supporting evidence in that the *Campylobacter* populations in the human and chicken hosts substantially overlap [7], [8], [9]. However, there is a wide diversity of *C. jejuni* strains in poultry flocks. These strains vary in both ability to survive the environmental stresses during processing and in putative virulence properties, such as invasiveness and toxin expression [10], [11]. Given this diversity it seems likely that many of these strains may be non-pathogenic *i.e.* not cause disease in humans. Either they lack virulence factors, do not colonize humans, or are not persistent during slaughter, processing and storage. Unfortunately, the virulence pathway or pathways by which *C. jejuni* causes disease are still enigmatic, not least because of the lack of a reliable animal model of disease [12].

The first complete genome sequence of a *C. jejuni* strain was published in 2001 for *C. jejuni* strain 11168 [13]. This laboratory-adapted strain was originally isolated from a case of campylobacteriosis in 1977. This strain displays a number of aberrant phenotypic characteristics, such as poor motility, an atypical straight body morphology, and poor colonization in chickens. In contrast a minimally-subcultured variant of the original clinical isolate displayed normal characteristics [14]. The virulence potential in humans of the sequenced variant is unknown, but other strains with impaired motility have been shown to have reduced virulence in a human volunteer study [15]. Genome sequences of other *C. jejuni* strains have since become publicly available, but for none of these sequenced organisms direct evidence exists of virulence in humans, or of transmissability from chicken to humans. For example, the sequenced *C. jejuni* strain 81116 [16] was originally isolated from a human case during a outbreak of campylobacteriosis in a school [17]. This outbreak was hypothesized to be due to the fecal contamination of drinking water by wild birds, but this was unconfirmed. Strain CG8486 is a recent clinical isolate, but has no stated epidemiological association with poultry [18]. In contrast, strain RM1221 was isolated from chicken [19], but its virulence potential in humans remains untested.

Following a research visit to a poultry abattoir, one surveillance team member developed campylobacteriosis. The strains isolated from the patient and from the poultry flock sampled at the abattoir were identical by serotyping, *fla* typing, pulsed-field gel electrophoresis, amplified fragment polymorphism and multilocus sequence typing (MLST), thus providing evidence of the direct transmission of pathogenic *Campylobacter* from a poultry source to a human.

The complete genome sequence of this strain, laboratory number 99/308, but designated here by its common name of M1, was determined in anticipation that its gene content would contain both colonization factors for chicken and virulence factors for human disease. With rapid advances in sequencing technologies and the subsequent explosion in the amount of available sequence data, it is now possible to describe microbial genomes not only as individual entities, but also to analyze their collective pan- and core genomes [20]. A core genome was first defined by Lan and Reeves (2000) [21] as comprising those genes present in almost all individuals of a species. The pan-genome was introduced by Tettelin and coworkers [22] as comprising any gene observed in the species. For defining the core and pan-genome, genes are considered members of a gene family when they share significant homology, using specified criteria. In this work we describe the comparison of the complete genome of *C. jejuni* M1 with other complete *C. jejuni* genome sequences.

## Results and Discussion

### Sampling of *C. jejuni* M1 and confirmation of chicken-to-human transmission

**Figure 1:**
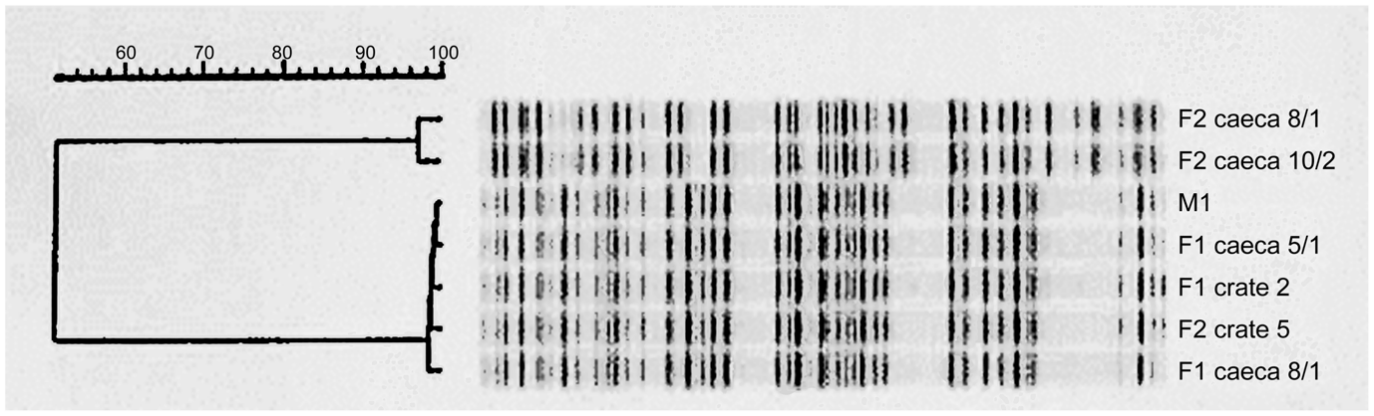
AFLP on strains isolated from abattoir. Isolates were from flock 1 (designated F1) and flock 2 (F2) and a selection of caecal isolates and crate swabs is shown. Two AFLP banding patterns were recognized of which the lower one is identical to that of the human isolate M1.

*C. jejuni* M1 (laboratory designation 99/308) was isolated from the diarrheic stools of one research team member 9 days after visiting a poultry processing plant. *C. jejuni* isolate M1 was found to be identical by all typing methods to isolates from samples from the processing plant derived from one flock (flock 1) but not from a second subsequent flock (flock 2) processed over the surveillance period. Figure 1 shows the AFLP patterns obtained. The strain persisted in the abattoir environment sufficiently to subsequently contaminate the crates and carcasses of the second flock processed (flock 2), indicating strong environmental survival properties.

### Genome sequence and characterization of *C. jejuni* M1

The human isolate *C. jejuni* M1, and epidemiologically related poultry strains are motile, S-shaped organisms with MLST Sequence Type (ST) 137, fla-type 2,5 [23], and serotype HS21. Additional characterization showed the strain to be without detectable plasmids. Further the M1 strain displays a low invasiveness to INT407 and CaCO2 cell lines [24] and to express low CDT activity as measured by *in vitro* assays (data not shown).

The genome sequence of strain M1 obtained from paired end reads, consisted of 18 contigs that were oriented using in-house perl scripts, and then assembled into a complete genome using directed PCR. To ensure the genome sequence corresponded with the human isolate, an *in silico* MLST was performed indicating Sequence Type 137 in accordance with the results obtained *in vitro*. An *in silico flaA* RFLP was also performed as a further confirmation (data not shown).

Gene finding predicted 1,624 protein encoding genes. Prediction of gene function, performed as described in the Methods, provided inferred functionality for 1,229 of these protein-coding genes. Non-translated genes coding rRNA or tRNA were also predicted. In addition to the expected three rRNA operons, strain M1 possesses 44 tRNA genes.

### Genes relating to invasiveness and colonization of chickens

A number of *Campylobacter* genes have been previously described as being related to chicken colonization and/or mediating adherence and invasion of human cells *in vitro*, on the basis of site-directed mutagenesis causing substantial loss of colonization or invasion potential. Most of these genes were present in strain M1. For example, *cadF,* which encodes the *Campylobacter* adhesion to fibronectin protein and is important in chicken colonization [25], [26], [27], *jlpA* [28] and *peb1A*, which encodes a 27-kDa putative adhesin [29] were identified as CJM1_1423, CJM1_0958 and CJM1_0885, respectively. In addition *porA*, which encodes the *C. jejuni* 43-kDa major outer membrane protein (MOMP), which is a porin and a potential adhesin [30], was identified as CJM1_1240. A putative adhesin Cj1279c, recently designated as fibronectin-like protein *A* coded by *flpA,* which has been shown as being required for efficient cell adherence and chicken colonization [31], was identified as CJM1_1260. Strain M1 also contains *ciaB*, a *Campylobacter* invasion antigen (CJM1_0879), the presence of which is reported to be associated with severity of campylobacteriosis in piglets [32]. Another putative *Campylobacter* invasion-associated gene [33], tentatively named *ciaC* but otherwise known as Cj1242, is identical to CJM1_1224.

**Figure 2:**
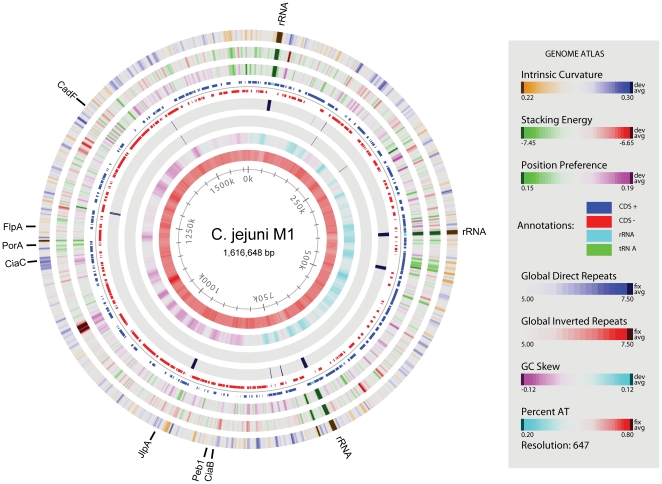
Genome Atlas of *C. jejuni* M1. The Atlas shows various nucleotide and structural properties as well as the genes of the M1 genome in relation to chromosomal position. The intrinsic curvature, stacking energy and position preference (a measure of helix flexibility, where green color denotes flexible sequences) are calculated from simple weight matrix models previously published [35], while global direct and inverted repeats are derived from BLASTN alignments [65]. The second inner-most circle displays the GC-skew, visible as the bias of G's towards the replication leading strand. Genes discussed in the text are marked on the atlas. A zoomable version is available online in the supplementary section.

Not all *C. jejuni* genes previously described as related to invasiveness were found in the genome of strain M1. Completely absent is *capA*, an autotransporter protein reported to be associated with both adherence to human epithelial cells and the colonization of chickens [34]. However, *capA* is known to be absent in many *C. jejuni* isolates [31]. The relationship between the absence of this gene and the phenotype of strain M1 is at yet unknown. A Genome Atlas of the strain M1 chromosome is shown in Figure 2. This Genome Atlas illustrates various features of the genome in eight circles [35], [36]. Based on the change in GC skew and on the presence of the *dnaA* gene, the first nucleotide of the sequence was positioned at the top of the circle as the likely origin of DNA replication. The fifth circle illustrates the annotated genes and reveals a slight preference for genes being located on the leading strand compared with the lagging strand. Around 1,050–1,100 kb there is a strong signal in the stacking energy lane, which suggests this region will readily melt, corresponding with the high local AT region mentioned previously. The functional annotation for this region inferred from Pfam indicates that genes in this area are involved in glycosylation and are responsible for biosynthesis of the lipooligosaccharide (LOS) structures of *C. jejuni*. The presence of readily melting DNA correlates with a higher AT content compared to the genome average and suggests that this region may be less thermodynamically stable and likely subject to an increased mutation rate [37]. The three rRNA operon loci are located in regions characterized by direct global repeats where the DNA is highly flexible, as shown from the green signal in the position preference lane, with an absence of protein coding genes. The flexible DNA in this region facilitates the binding of the RNA polymerase and a high expression level for these genes.

### Core and Pan-genome Analysis

Campylobacters have a relatively small genome, which has implication for the sizes of the pan- and core genomes. With only roughly 25–33% as many genes as *E. coli* strains, such small genomes would be expected to contain more essential (and hence ‘core’) genes and relatively fewer dispensable or auxiliary genes [38].

An estimate was made of the pan- and core genomes of *C. jejuni* based on the 13 complete or nearly complete genomes (including M1) of *C. jejuni* publically available at the time of writing. The core genome of the genus *Campylobacter* could be estimated tentatively using 8 additional genomes of other *Campylobacter* species, although more data would allow for higher accuracy. The core genomes for each of the *non-jejuni* species could not be assessed, however, as only single genome sequences for each species were available.

**Figure 3:**
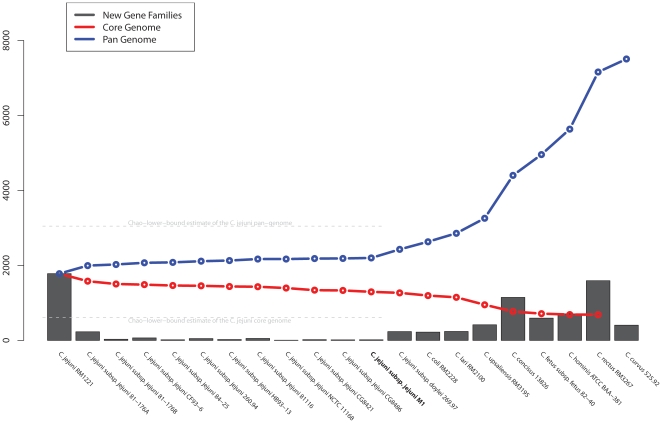
Pan-genome plots of *Campylobacter*. The columns give the number of new gene families introduced with the addition of each new genome to the consideration. The curves shows the evolution of the pan genome as an accumulated sum of gene families (blue line) and the core genome (red line), as described in the Methods. The lower-bound estimates of the sizes of *the C. jejuni* core and pan-genomes are indicated by the dotted lines.

The number of conserved gene families in the core and pan-genomes of the genus *Campylobacter,* identified by consecutive addition of species, starting with the *C. jejuni* genome containing the most genes (*C. jejuni* RM1221) and then subsequently adding the remaining *C. jejuni* genomes one by one, is shown (Figure 3). The resulting numbers of newly added gene families were plotted as bars, whereas the lines connect the sum of all recognized gene families (the pan-genome) and the combined conserved gene families (the core genome).

With the addition of each genome, the size of the pan-genome increases, whilst the core genome decreases at a slower rate. In total 2,427 gene families were identified in the pan-genome whilst the core genome comprised 1,295 gene families. Note that in **Figure 3**, the genome of strain M1 reduced the estimated core genome of *C. jejuni* by 6 gene families, but contributed 16 new gene families to the pan-genome. Based on the 13 *C. jejuni* genomes available, and using a method previously described [39] for extrapolating the boundaries of core and pan-genomes, we estimate the total species core genome to stabilize no lower than 608 gene families. A similar Chao-lower-bound estimate of the pan-genome size is 3,047 gene families which represents the minimum number of gene families one would expect to see with infinite data available [40]. These numbers were inferred using a strict regimen requiring core genes to be present in all 13 genomes and should be considered the extreme lower boundaries for the core and pan-genomes. Two recent studies reported an estimate number of 647 or 847 for the core genome of the complete genus *Campylobacter* using different approaches [40], [41]. A similar estimate for the genus using the method applied here would not be justified with the amount of data available.

The pan-genome of all 13 *C. jejuni* genomes analyzed is roughly twice that of their core genomes, and only about 1.5 times the average size of these 13 *C. jejuni* genomes. Consequently only about one third of any of these genomes will comprise auxiliary genes (*i.e.* genes not belonging to the conserved core genome) and these must then account for strain-to-strain phenotypic variation that is due to gene content (as opposed to variations due to gene expression or the minor sequence variations possible while remaining inside the same gene family). Such genes may be functionally dispensable and redundant to some degree. However, it is not known if all such genes would be non-essential as a given ‘essential’ functionality could theoretically be provided by different auxiliary genes in alternative genetic backgrounds.

**Figure 4:**
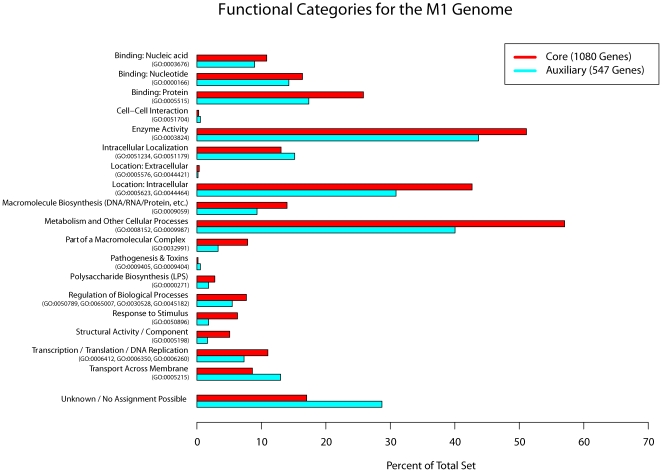
Gene Ontology terms associated with core and auxiliary genes. The figure shows the percentage of the total core and auxiliary genomes for strain M1 which could be associated with a given role based on Gene Ontology terms. The Gene Ontology terms used to connect each protein to a functional category is given in parenthesis below each category. The total fraction of each set which could not be assigned is also presented.

Functional categories for the core and auxiliary genomes for *C. jejuni* M1 were inferred through alignments of translated sequences against UniProtKB and the results were summarized in **Figure 4**. For the core genes a greater focus can be seen on intracellular activities exemplified directly by the higher fraction of the core genome being associated with categories like “Location: Intracellular”, “Metabolic and other Cellular Processes” and “Transcription/Translation/DNA Replication”. The Auxiliary genes were generally harder to assign to a category, but show a higher degree of involvement in transporting molecules in or out of the cell. Even so, a large fraction of the auxiliary genes are still connected with fundamental cellular and metabolic processes.

**Figure 5:**
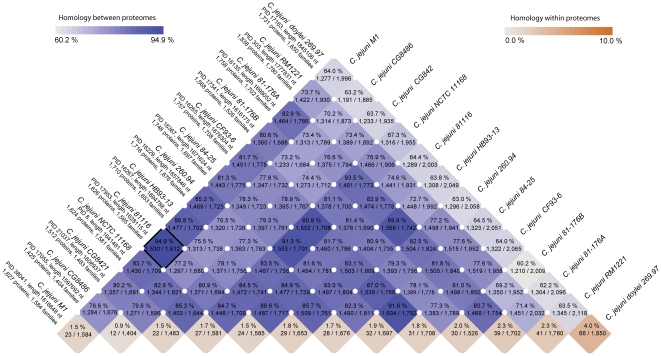
Blast Matrix of *C. jejuni.* The figure presents the absolute number of gene families preserved between any two species along with the total number of families between them. The relative percentage between these numbers is also given and is used as a basis for the color intensity. The orange squares deserve special mention since these are based on alignments of the organism against itself, and thus show the internal homology within each organism's proteome. The highest homology was found between genomes M1 and 81116, highlighted by a box.

An alternative comparison of all genomes was undertaken, based on a matrix of an all-against-all BLAST analysis (**Figure 5**). This provides an overview of how similar any genome is to any other genome, in terms of the number of conserved gene families. On the basis of this analysis the genome displaying the highest degree of similarity to strain M1 is that of strain 81116. As expected, the least degree of similarity of any single genome to all of the other genomes analyzed here is reported for *C. jejuni doylei* 269.97.

### Clustering of *Campylobacter* proteomes

**Figure 6:**
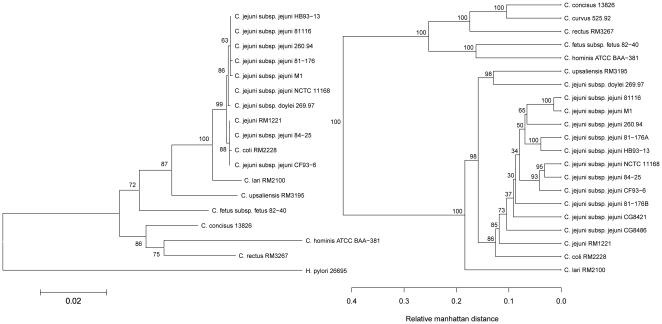
16S rRNA and Pan-genomic tree of the *Campylobacter* genus. The left dendrogram displays the distance between the genomes based on the sequence of the 16S rRNA gene. The right dendrogram is based on presence or absence of the gene families in the pan-genome. The relative manhattan distance indicates the proportion of the pan-genome where genomes differ in present/absent status. Bootstrap values are given as percentages to indicate the stability of the branching.

A pan-genome hierarchical clustering tree based on the gene family content, along with a phylogenetic tree based on 16S rRNA similarity was generated (**Figure 6**). The pan-genome tree is an alternative presentation of the data previously shown in **Figure 3** and **Figure 5**, but emphasizes inter-species relationships. As expected, genomes reporting a high similarity score in the BLAST matrix cluster on the pan-genome tree, which provides deeper resolution among the *C. jejuni* genomes than the 16S rRNA tree. The close relationship of the single *C. coli* genome with all the *C. jejuni* strains is apparent, while the *C. jejuni* subspecies *doylei* strain is an outlier to the other sequenced *C. jejuni*, corroborating its dissimilarity to the other genomes in the BLAST Matrix of Figure 5. In fact, while the 16S rRNA profile places the *doylei* subspecies within the *C. jejuni* clade, the pan-genome tree reveals its gene content to be closest to that of *C. upsaliensis*. While the gene content alone is no evidence of evolutionary or taxonomical relationship, it does suggest that *C. jejuni doylei* may display some functional characteristics normally only associated with *C. upsaliensis* and not other *C. jejuni*. Indeed, *C. jejuni doylei* is typically not encountered in food or animals, but only in humans, where it appears more apt at causing bacteremia [42], suggesting a different ecology.

The high degree of whole-genome similarity between strains M1 and 81116 is confirmed by the relative Manhattan distance being less than 0.05, *i.e.* these strains differ in less than 5% of the pan-genome (ignoring all ORFans, see Methods).

### Comparison of 81116 and M1

*C. jejuni* strain 81116 is the closest related sequenced strain to M1. It is known to be serotype HS6, ST-283 (which differs in 3 of 7 alleles with strain M1). Although strain 81116 has the same AFLP profile as strain M1 (data not shown), its DNA is indigestible by *Sma*I so it is untypable by PFGE using this enzyme [43]. Strain 81116 is one of a group of strains that were isolated over time and distance with identical genotypes [43] and consequently share phenotypic characteristics. A closer comparison of M1 with strain 81116 was undertaken.

The frequency of genes sharing decreasing percentages of identity between these two genomes was determined (**Table 1**). These data illustrate the differences that can occur, on a complete genome scale, between isolates that have an identical AFLP genotype and *flaA* type. The divergence in gene content between these strains is considerable, even at the protein level. Such observations would obviously be overlooked when isolates were only characterized by genotyping, and this could lead to the erroneous interpretation that isolates with identical genotypes are completely identical.

**Table 1:** Genes identified in a comparison between strains 81116 and M1 as unique relative to the comparator.

	Number of Proteins	
BLASTP %-identity	M1 proteome (1624 proteins) vs. 81116 proteome	81116 proteome (1623 proteins) vs. M1 proteome
100%	1166	1194
≥90%	392	322
≥80%	23	19
≥70%	7	13
≥60%	6	8
≥50%	2	11
≥40%	0	4
≥30%	4	5
≥20%	5	11
<20%	19	36
	Proteins with <20% identity in the other genome	
	*CJM1_0032, CJM1_0040*, *CJM1_0041*, *CJM1_0055, CJM1_0056, CJM1_0057, CJM1_0138, CJM1_0278*, *CJM1_0414, CJM1_0728, CJM1_0745*, CJM1_1118, *CJM1_1119,* CJM1_1120, CJM1_1372, CJM1_1373, *CJM1_1375*, CJM1_1381, CJM1_1382	*C8J_0035, C8J_0036, C8J_0063, C8J_0132, * ***C8J_0133*** *, C8J_0154, C8J_0265, C8J_0266, C8J_0267, C8J_0272, * ***C8J_0273, C8J_0274*** *, * ***C8J_0275,*** * C8J_0321, C8J_0529, C8J_0651, C8J_0776, C8J_0852, C8J_0918, C8J_1080, C8J_1081, C8J_1083, C8J_1329, C8J_1330, C8J_0131, C8J_1332, C8J_1334, C8J_1335, C8J_1337, C8J_1338,* *C8J_1340, C8J_1341, C8J_1342, C8J_1456, C8J_1458, * ***C8J_1459***

*Gene names given in bold refer to the sequences having matches to verified proteins in UniProtKB/Swiss-Prot, while italics indicate a match to the NCBI nr database.

In the comparison of the genomes of strains 81116 and M1, a number of genes were recognized as being unique to either one or the other strain (**Table 1**). The comparison was performed first with a tBLASTX alignment of the genes of *C. jejuni* M1 against the full genome of 81116 and then *vice-versa*. Similarity was relaxed to require 50% identity over only 20% of the length of the query sequence (*i.e.* <10% identity over the entire sequence), so that only genes that are truly missing (as opposed to divergent genes) in either of the two strains were detected. There were 36 genes unique to strain 81116 and 19 unique to strain M1. One of the genes present in strain 81116, but not in strain M1, was C8J_0133, which has similarity to a DNA methylase in *Lactococcus lactis* subsp. *cremoris* (P34877). The absence of this gene might explain the resistance to *Sma*I digestion observed strain 81116 but not in strain M1.

### Blast Atlas of *C. jejuni* strain M1

**Figure 7:**
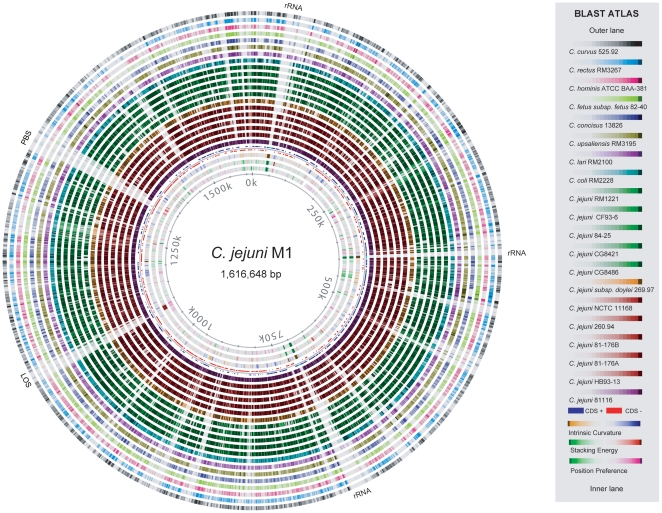
Blast Atlas of *C. jejuni M1*. The proteome of the M1 strain was aligned against the proteomes of 20 other *Campylobacter* genomes using BLASTP and the results are displayed as colored circles with increasing color intensity signifying increased similarity. Only BLAST results of proteins are shown. The three rRNA islands are marked as well as the lipooligosaccharide (LOS) and extracellular polysaccharide biosynthesis (PBS) clusters.

A final visualization tool of genome comparison applied to the *C. jejuni* genomes was the Blast Atlas [44] (**Figure 7**) (a “zoomable” version of this is available online [45]). This analysis used the Genome Atlas of M1 as a reference point, around which all BLAST hits detected in the other *C. jejuni* genomes were plotted, followed by the other available genomes of the *Campylobacter* genus. The atlas illustrates how well conserved most regions of strain M1 are within the other *C. jejuni* species, and even to a large extent within the *Campylobacter* genus, with the exception of a few loci. As this analysis is based on BLASTP, RNA genes show up as ‘gaps’ although they are in fact conserved in all genomes.

Despite the degree of conservation, several highly variable regions are also visible, such as the gene cluster located in the region from 1,335 kb to 1,360 kb. This region encodes several genes which are hypothetical, but also many capsular polysaccharide biosynthesis genes. The LOS locus of *C. jejuni* also displays high degrees of variability between strains, as previously reported [13].

At the position from 52,774 to 51,104 bp the gene CJM1_0038 is located. This gene encodes a gamma-glutamyltranspeptidase, which has been shown to enhance the ability of *C. jejuni* to colonize the avian intestine [46], [47]. This gene is far from ubiquitous in *C. jejuni* but is found (other than in M1) in strains 81116, HB93-13, 81-176 and *C. jejuni* subsp *doylei* 269.97. Although all these strains were isolated from cases of campylobacteriosis [16], [48], other clinical strains, such as NCTC 11168, do not possess this gene.

Another region of interest, from 1,455 kb to 1,465 kb, encodes two Type I restriction modification genes *hsdR* and *hsdS* (CJM1_1490 and CJM1_1493) and the associated DNA methylase, *hsdM,* (CJM1_1494). This restriction/modification (R/M) region has been shown to be involved in a *hsd* type I restriction–modification system of *C. jejuni* but is not present in *C. jejuni* strain NCTC 11168 [49]. The region appears conserved in only a few *C. jejuni* strains in addition to M1, namely in strains 81116, CG8421, and GC8486, as well as in *C. coli* RM2228. While *hsdR* and *hsdM* were found conserved in strain HB93-13 the *hsdS* gene was not. The presence of this R/M region would suggest implications for the methylation pattern and susceptibility of these genomes towards the HsdR restriction enzyme.

Finally, the R/M region in strain M1 also contains two slightly overlapping genes, *rloA* and *rloB* (CJM1_1491 and CJM1_1492) with no inferred function, but which are also conserved in strains 81116 and CG8421. A subsequent examination of these genomes revealed that the location of the *rlo* genes inside the R/M island is conserved in strains CG8486 and 81116 (data not shown).

In conclusion, strain M1, a rarely reported *C. jejuni* isolate from colonized poultry with direct evidence of its ability to infect and cause diarrhea in a human, provides a unique opportunity to investigate the genomic basis of colonization in poultry and pathogenicity in humans. The complete elucidation of the genome sequence of strain M1 not only provides the scientific community with a valuable strain to use in *in vitro* and *in vivo* models of colonization and virulence, but also allows *in silico* approaches such as microarray analysis to be applied.

## Materials and Methods

### Ethics Statement

The study was approved by the VLA Ethical Review Committee on the 25th of March 2010. The patient has provided written informed consent for the collection of samples and subsequent analysis.

### Isolation and characterization of *C. jejuni* strain M1

A research team of four individuals visited a poultry processing plant to investigate the sites and diversity of *Campylobacter* contamination. Three days after this visit, one member of the research team became ill with watery diarrhoea, fever and severe abdominal cramps. The diarrhoea terminated after about six days. Six days after the onset of symptoms a faecal sample was cultured as described below and *C. jejuni* was isolated. Four separate colonies from the faecal sample were recovered and stored.

The team sampled two flocks (flocks 1 and 2) at the abattoir. The flocks were reared on different farms, and sequentially processed through the processing plant. Caeca were removed from the poultry carcasses during evisceration, and the contents sampled aseptically. Swab samples were also collected from various sites in the abattoir and from up to five poultry carcasses [50] as they progressed through the processing plant. The methods of chicken sampling and culture have been previously described [51]. Briefly all samples were directly plated onto blood agar containing selective antibiotics [52] with actidione (100 ug/ml) and cefoperazone (30 mg/ml), incubated microaerobically at 37°C for 2 days, with or without pre-enrichment in Exeter medium [53]. Identification was based on microaerobic growth at 42°C, hippurate and indoxylacetate hydrolysis and catalase and oxidase activities. A single colony from each sample was stored in glycerol broth (10% v/v glycerol in 1% w/v proteose peptone) at −80°C for subsequent typing.

All isolates were *fla*-typed according to the technique of Ayling et al. [23] using the restriction enzymes *Dde*I and *Hinf*I. Representatives of the human and chicken isolates were also serotyped [54] and molecular typed by pulsed-field gel electrophoresis (PFGE) using the enzymes *Sma*I and *Kpn*I [55], amplified fragment length polymorphism (AFLP) [56] and multilocus sequence typing (MLST) [57].

Phenotypic characterization of strain M1 (Laboratory designation 99/308) was performed to determine *in vitro* invasiveness of INT407 cells and CaCO2 cells and to detect the expression of cytolethal distending toxin (CDT) as previously described [58], [59].

### Genome sequencing and annotation of strain M1

The genome of strain M1 was sequenced by Agencourt Bioscience, Beverly MA, USA using a 454 GS FLEX instrument and assembled using the ARACHNE system of Batzoglou *et al* [60]. This resulted in 18 contigs ranging in size from 108 bp to 950,427 bp, altogether totaling 1,611,563 bp. Gaps were closed by direct sequencing of PCR amplifications using primers flanking the gapped regions. The gene annotation (as described below) for strain M1 was visualized on a Genome Atlas as previously published [35], [36]. A zoomable version of the Atlas is available online [45]. The complete annotated sequence has been submitted to NCBI/GenBank under the accession number CP001900 (Genome Project ID 38041).

### Identification of protein coding genes and inference of gene function

Gene finding was carried out using the EasyGene v1.2b gene finder using the pre-calculated model for *C. jejuni* (CJ02) [61], [62], which resulted in 1624 inferred protein coding genes. The quality of the gene finding is difficult to gauge, but was found to be acceptable in an assessment performed according to Skovgaard *et al.* [63] (See supplementary section [45]). Even so, in their second publication, the authors of EasyGene note that the gene finder has a small tendency to disfavor non-ATG start codons, particularly if an in-frame ATG is nearby [61]. Thus, to verify the correct prediction of CDS start codons, all inferred genes were aligned against the NCBI non-redundant database (nr database) using BLASTX [64], [65], which identified 233 entries as having no perfect match in the database. For each of these 233 entries, the highest scoring sequence in the nr database was extracted and aligned back against the entire strain M1 genome using TBLASTN. If this resulted in a perfect hit to the sequence of the originally predicted gene in strain M1 covering the entire sequence in the nr database without any in-frame stop codons, the strain M1 annotations were updated. In this case a new transcription start position was assigned to the M1 gene matching that of the sequence from the nr database, provided that the new start codon was a valid. As a result of this, 85 start codons were changed, many of which resulted in non-standard, but valid start codons of GTG or TTG. A table of all affected genes is available in the supplemental section [45].

Functional information was inferred through prediction of gene function. This entailed the comparison of translated sequences to the validated part of UniProt (Swiss-prot) [66], and to the annotated genes of the previously sequenced Campylobacter genomes in GenBank (**Table 2**) [67]. For BLASTX alignment a similarity criterion was used of ≥50% identity of the amino acids over ≥50% of the length of the longest sequence. Hits to Uniprot were preferred over those to the NCBI database, however, the curated Swiss-prot part of Uniprot was preferred over all others. In total, similarity to Swiss-prot provided inference of annotation for 527 protein coding genes, while a further 1060 could be characterized from the rest of Uniprot (UniProt/TrEMBL) and 8 from NCBI nr. Of these 1595 genes, 495 were annotated as “putative”, “uncharacterized” or “hypothetical”.

Supplemental functional annotation was inferred through the alignment of translated sequences against active sites in Pfam using the HMM-based tool provided by Pfam [68]. Default settings were used and the built-in Pfam TC trusted threshold cutoffs were relied upon. In case of overlapping results, only the best match was used.

### Identification of non-protein encoding RNAs

Ribosomal genes coding rRNA were predicted using RNAmmer [69], while tRNA genes were predicted using tRNAscan [70]. Both programs were used at the default settings.

### Identification of gene conservation and definition of gene families

The *Campylobacter* genomes used for comparative studies, as listed in **Table 2**, were obtained from NCBI [67].

The set of gene families for this collection of *Campylobacter* genomes were found by BLASTing all proteins in each genome against all proteins in the query genome. A hit was considered significant if the alignment covered at least 50% of both sequences, and contained at least 50% identities [71]. This included aligning proteins within each proteome, and while self-hits were ignored, hits to other homologous genes within the same genome were recorded. A pair of genes having significant hits was considered as belonging to the same gene family. Two gene families were merged into one if any one gene in a given family would meet the similarity criterion in an alignment against any member of another gene family [71], [72].

### BLAST matrix

The results of the gene conservation analysis were visualized as a triangle-shaped matrix providing the amount of conserved gene families between any two proteomes, both as an absolute number and as the fraction of the total number of gene families shared. In addition to the comparisons across proteomes, the matrix also shows the results from aligning proteins within each proteomes against each other.

### 16S rRNA tree for the *Campylobacter* genus

RNAmmer was used to find the sequences for the 16S rRNA phylogenetic tree. The sequences had to be between 1400 and 1700 nucleotides and have an RNAmmer score above 1700 to be chosen. In case several sequences from one organism satisfied these criteria, one sequence was arbitrarily chosen. Alignment was done using PRANK [73], [74], and the program MEGA4 was used to construct a phylogenetic tree [75]. Within MEGA4, the tree was created using the Neighbor-Joining method with the uniform rate Jukes–Cantor distance measure and the complete-delete option. A thousand re-samplings were done to find the bootstrap values.

### Pan-genome tree for the *Campylobacter* genus

The pan-genome tree is another method for visualization of the differences between proteomes in a pan-genome context. It is a hierarchical clustering of genomes based on a weighted Manhattan distance computed from a pan-matrix. A pan-matrix is a matrix of 1s and 0s where each row corresponds to a gene family, as described above, and each column to a genome. Cell *(i,j)* in the matrix is 1 if gene family *i* is present in genome *j*, or 0 if it is absent. The distance between two genomes is the proportion of gene families where their present/absent status differs. Shorter distances represent genomes with many gene families in common, and larger distances reflect genomes with fewer gene families in common. Genes only occurring in a single genome were given a weight of “0” – that is, they did not provide any difference in the computed distance in the matrix. These ‘ORFans’ are frequently annotated as ‘hypothetical protein’ and may in cases be falsely predicted genes. By discarding these genes the tree obtained is more robust against gene-prediction errors, at the cost of possibly losing some information. Bootstrap values (percentages) were computed for each inner node by re-sampling the rows of the pan-matrix.

### Core- and pan-genome analysis

Conservation data across various genomes were also visualized by an accumulative plot showing the changes to the number of gene families in the core and pan-genomes as more sequential genomes were added to the dataset [76]. The number of novel gene families for each added genome is also depicted. Any novel gene family encountered is automatically added to the pan-genome unless it is found to be conserved in the previously considered genomes. Because the quality of genome annotation is not consistent, and because **Table 2** includes genomes which have not been completely sequenced, we have relaxed the definition of the core genome as follows; we added any gene family of the n^th^ analyzed genome to the core genome if by analyzing n+1 genomes this gene family was present in at least n genomes. This approach provided tolerance while preserving intuitiveness in plotting. Otherwise, the reduction in the core genome caused by, for example, the addition of *C. concisus* 13826 would be drawn for the genome to its right (*C. fetus*). For the same reason the core genome curve does not extend to the rightmost genome since no n+1 genome exists.

GO categories were inferred for the core and auxiliary genes in strain M1 through the alignment of translated sequences against Uniprot meeting the similarity criterion of at least 50% identity covering at least 50% of both sequences. GO terms were inferred from all alignments meeting the criterion to minimize problems with incomplete annotations in the database. For each go term assigned to a given protein, all parent terms were considered as well. GO assignments were then grouped together into functional category based on similarity and overlap.

### BLAST atlas

A Blast Atlas was produced using a previously described approach [44]. The atlas displays results of the BLASTP comparisons of the products of the predicted genes of *C. jejuni* M1 with the annotated proteomes of the other campylobacters from **Table 2**. A zoomable version is available online [45].
